# Supplementary material for: Nanofibrous Vildagliptin/PLGA Membranes Accelerate Diabetic Wound Healing by Angiogenesis
Source: Pharmaceuticals (Basel). 2022 Nov 4;15(11):1358. doi: 10.3390/ph15111358 (PMC9696371; doi:10.3390/ph15111358)
Supplement: Supplementary file 1 [file pharmaceuticals-15-01358-s001.zip › pharmaceuticals-1982469-supplementary.pdf]

Nanofibrous Vildagliptin/PLGA Membranes Accelerate Diabetic Wound Healing by Angiogenesis

* To whom correspondence and reprint requests should be addressed:
Chen-Hung Lee, E-Mail: chl5265@gmail.com
Chi-Ching Kuo, E-Mail: kuocc@mail.ntut.edu.tw
Shih-Jung Liu, Email: shihjung@mail.cgu.edu.tw

List of Supplementary Information:
Table S1. Variations in water content in the fabricated nanofibrous membranes over 24 h.
Figure S1. FTIR spectra of PLGA and vildagliptin/PLGA nanofibrous membranes.

Table S1. The variations in the water content over 24 h.

Time (Hour)	Vildagliptin Group	Control Group	*p*-Value
0.5	260 ± 30	49 ± 19	<0.001
1	340 ± 24	39 ± 18	<0.001
2	380 ± 38	41 ± 36	<0.001
3	520 ± 69	19 ± 7	<0.001
8	358 ± 24	19 ± 10	<0.001
24	367 ± 30	33 ± 13	<0.001

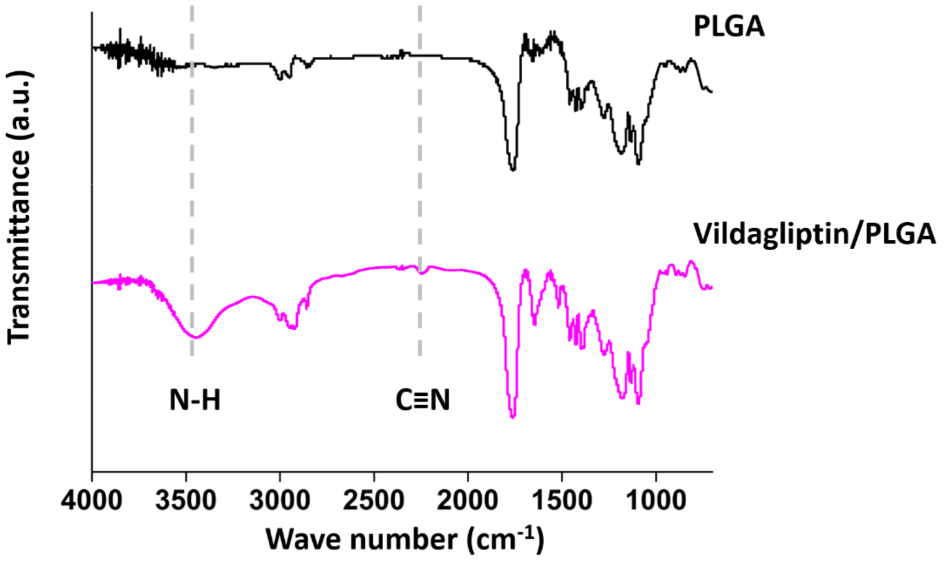

Figure S1. FTIR spectra of PLGA and vildagliptin/PLGA nanofibrous membranes.
